# Editorial: Integrated health service delivery and COVID-19

**DOI:** 10.3389/fpubh.2022.1008777

**Published:** 2022-09-15

**Authors:** Brijesh Sathian, Edwin van Teijlingen, Padam Simkhada

**Affiliations:** ^1^Geriatrics and Long Term Care Department, Rumailah Hospital, Hamad Medical Corporation, Doha, Qatar; ^2^Department of Midwifery and Health Sciences, Faculty of Health and Social Sciences, Bournemouth University, Poole, United Kingdom; ^3^Department of Allied Health Professions, Sport and Exercise, School of Human and Health Sciences, University of Huddersfield, Huddersfield, United Kingdom

**Keywords:** health-care systems, COVID-19, integrated health service delivery, coordinated care, infection control, willingness, health policy

## Introduction

The coronavirus (COVID-19) pandemic has created significant economic, political, medical, and societal concerns around the world. The COVID-19 pandemic has brought a lot of mayhem to health systems across the globe. It has forced politicians, policymakers and health service managers to reconsider the way we organize both individual health services and our whole health system. Promoting integrated health-care delivery systems is one of these considerations. Integrated health services emphasize capability building in primary care, recruiting more qualified family doctors, improving infrastructure, and improving coordination between tertiary hospitals and community health centers. The World Health Organization (WHO) defines integrated care as “the organization and management of health services so that people get the care they need, when they need it, in ways that are user-friendly, achieve the desired results and provide value for money” (1). Relocating more healthcare resources and patients into the community will help to transform the present hospital-centric healthcare delivery system and increase access to healthcare and whilst making it more affordable.

An essential goal in advanced healthcare delivery is integrated care to combat health system decentralization and providing attentive patient-centered treatment (2). It has been consistently identified as a priority for health services reform to ensure health systems can meet patient needs while managing rising costs (3, 4). Integrated care has also been recognized as an important mechanism by which to achieve the quadruple aim of health system optimization: (a) enhancing patient experience; (b) improving population health; (c) reducing costs; and (d) improving the work life of health care providers (5).

How health systems throughout the world respond to future pandemics or even national epidemics is likely to effect not just how healthcare is delivered but also how policymakers, healthcare workers, and society as a whole perceive such crises. A key element of obtaining universal health coverage is an appropriate integrated, people-centered health system. The need of integration in creating resilient health systems that can endure the impact of medical emergencies has also been emphasized.

Service delivery, health workforce, medication, and technology were the most often integrated health system building elements during the COVID-19 pandemic, according to a scoping review by Hasan et al. (6). Low and Middle-Income Countries (LMICs), particularly the poorer nations, primarily adopted the integrated health service delivery system through systematic horizontal integration, driven by particular policy measures, as a response to COVID-19. The key enabler for integrated health-care systems was the government's supervision, together with local institutions' decentralized decision-making abilities and multisectoral cooperation. The main obstacles to integration were concurrently scattered service delivery systems, a weak supply chain, limited diagnostic capacity, and a shortage of human resources.

It is vital to recognize and take lessons from health system measures that have been utilized to stop the spread of COVID-19. When the COVID-19 pandemic evolves, knowing this can help with decision-making about the planning, financing, structure, and administration of health services. On the global synergy between integrated health care and COVID-19 pandemic readiness, there is a severe lack of research, and hence comprehensive evidence synthesis. There were four papers in this Research Topic that described the experiences of from four different countries from across the globe.

Kim and Rao study reveals that the State of Michigan (USA) including the city of Detroit, could not reach herd immunity given the slow progress of vaccination efforts. Although Detroit's incentive scheme increased the weekly vaccination rate by 44.19% for the first dose (from 0.86 to 1.25%), it was ineffective in improving the rate of uptake of the second dose. The authors raise the importance of the incentive scheme in the light of the rather limited access to certain services, especially transport for vulnerable people such as the poor and elderly or localities where the public transit infrastructure is poor. This study findings will be useful to the policymakers to implement vaccination incentive schemes to increase vaccination rates in regions where current rates are still insufficient to achieve herd immunity.

A study by Kirchberg et al. shows that the impact of the COVID-19 pandemic on the presentation of oncological patients in a CCC (comprehensive cancer center) in Germany was considerable. The number of presentations decreased by 3.2% during the COVID-19 year 2020 compared with the pre-COVID year 2019 and more pronounced by 9.4% during the first lockdown from March to May 2020 compared with March to May 2019. This decrease was significant for curable cases of esophageal cancer and colon cancer as well as biliary tract cancer during the first shutdown from March to May 2020. Targeted countermeasures should be taken to avoid disastrous effects on oncological patients' access to vital services, as the pandemic is still ongoing.

In a Qatari facility for geriatric care, a COVID-19 epidemic was successfully contained, according to research by Al Hamad et al.. The study concluded that there was still evidence of inadequate infection control procedures, such as breaking infection control rules, including using personal protective equipment improperly, despite all the pre-existing preventative measures that were put in place at the beginning of the pandemic. The epidemic was effectively managed by the infection prevention and control team with promptly reassessed and implemented more stringent infection control methods and practices. The common comorbidities were dementia followed by coronary artery disease, diabetes mellitus, and seizures. Older adults of long-term care facilities are more likely to end up with more COVID-19 symptoms, because they have specific pre-existing comorbidities. Thus, for successful outbreak prevention and treatment, intense infection control measures such constant staff compliance monitoring, patient and caregiver monitoring, visitor policy amendment, prompt contact tracing, and continual education and awareness raising are key recommendations.

Our fourth research study was conducted by Liu et al. investigating the willingness for COVID-19 vaccination among Chinese medical students, which showed a sub-optimal trend. The readiness to get the COVID-19 vaccination was significantly associated with attitudes about the levels of eHealth Literacy, the impact of COVID-19, worries about the vaccine, and gender.

We believe that an evidence-based strategy, especially in a pandemic like the current COVID-19 one, can help address issues around delivering better, cost-effective, cutting-edge, and comprehensive services that begin to fit the definition of integrated health care.

## Author contributions

BS has prepared the manuscript draft, while EvT and PS have revised it for important intellectual content. All authors contributed to the article and approved the submitted version.

## Conflict of interest

The authors declare that the research was conducted in the absence of any commercial or financial relationships that could be construed as a potential conflict of interest.

## Publisher's note

All claims expressed in this article are solely those of the authors and do not necessarily represent those of their affiliated organizations, or those of the publisher, the editors and the reviewers. Any product that may be evaluated in this article, or claim that may be made by its manufacturer, is not guaranteed or endorsed by the publisher.
